# The prevalence of alcohol dependence and its association with hypertension: a population-based cross-sectional study4 in Xuzhou city, China

**DOI:** 10.1186/s12889-018-5276-1

**Published:** 2018-03-16

**Authors:** Ailing Ji, Peian Lou, Zongmei Dong, Chunrong Xu, Pan Zhang, Guiqiu Chang, Ting Li

**Affiliations:** 1 Department of Internal Neurology, Xuzhou Third People’s Hospital, 131 Huancheng Road, Xuzhou, China; 2 Department of Control and Prevention of Chronic Non-communicable Diseases, Xuzhou Center for Disease Control and Prevention, 142 West Erhuan Road, Xuzhou, Jiangsu 221000 China; 3 School of Public Health, Xuzhou Medical University, 209 Tongshan Road, Xuzhou, Jiangsu 221000 China

**Keywords:** Alcohol dependence, Hypertension, Cross-sectional study

## Abstract

**Background:**

To describe the prevalence of alcohol dependence and to explore the relationship between alcohol dependence and newly detected hypertension in China.

**Methods:**

A multistage stratified cluster sampling method was used to obtain samples from February to June 2013. The Michigan Alcoholism Screening Test was used to estimate alcohol dependence level. A standard questionnaire measured other independent variables. Enumeration data were analyzed using chi-square; quantitative data were analyzed using t-tests. Spearman correlation analysis and multivariate logistic regression analysis were performed to identify the relationship between alcohol dependence and hypertension.

**Results:**

The alcohol dependence rate was 11.56%; 22.02% of males (3854/17501) and 1.74% of females (324/18656) were classified as alcohol dependent. The newly detected hypertension rate was 9.46% (3422/36157). Significant associations were found between alcohol dependence levels and blood pressure (*P* < 0.01). Alcohol dependence was positively correlated with systolic blood pressure (*r* = 0.071, *P* < 0.01) and diastolic blood pressure (*r* = 0.077, *P* < 0.01) and was an independent risk factor for hypertension after adjusting for confounders (low alcohol dependence: odds ratio [OR] = 1.44, 95% confidence intervals [CI] = 1.14–1.81, *P* < 0.01; light alcohol dependence: OR = 1.35, 95% CI = 1.11–1.64, *P* < 0.01; medium alcohol dependence: OR = 1.83, 95% CI = 1.40–2.41, *P* < 0.01).

**Conclusion:**

Alcohol dependence was high and associated with hypertension. Health education and precautions against alcoholism should be implemented in Xuzhou city.

## Background

Alcohol dependence involves a range of behavioral, cognitive and physiological symptoms, of which the main characteristic is “preoccupation with alcohol overriding other interests, persistent drinking despite obvious harm and withdrawal symptoms on stopping” [1]. Alcohol dependence plays a major role in the relationships among alcohol consumption, illness and mortality [2] and has an estimated prevalence of 5.0% in 6 European countries [3]. The effects of alcohol dependence contribute substantially to the global burden of disease: 4.1% of global disability-adjusted life-years is attributed to alcohol [4], and the annualized death rates for alcohol dependence are 4.6-fold higher for women and 1.9-fold higher for men compared with the general population [5]. Patients who are alcohol dependent are usually more socially marginalized and have higher rates of physical comorbidity [3]. Additionally, substantial barriers exist to the implementation of effective options to treat alcohol dependence in primary care [6]. Thus, alcohol dependence is the third most serious public health problem, only surpassed by cardiovascular diseases and malignant tumors [7].

Alcohol consumption has a causal impact on a number of diseases, including several types of cancer, ischemic heart disease, ischemic and hemorrhagic stroke and liver cirrhosis [8]. Quintana and colleagues [9] have found that alcohol dependence is associated with reduced heart rate variability, which may predict adverse future outcomes, including cardiovascular disease and sudden cardiac death [10]. Of these cardiovascular diseases, hypertension is a leading cause of death [11]. Approximately 1.56 billion people have hypertension or related symptoms [12]. According to Taylor et al. [13], the effect of alcohol consumption on hypertension is entirely detrimental and characterized by a dose–response relationship that shows a linear increase of relative risk with alcohol consumption. However, research has mainly focused on alcohol consumption rather than alcohol dependence. Additionally, the literature on the relationship of alcohol dependence and hypertension shows conflicting results. Some studies have found an association between alcohol dependence and increased hypertension risk [14], whereas others have found converse association between alcohol dependence and hypertension [15]. Moreover, most studies have been carried out in Western countries, so the results cannot be extrapolated to broader populations. What are the epidemiological distribution and status of alcohol dependence in China? Is alcohol dependence associated with hypertension in the Chinese population? The primary aim of this cross-sectional study was to assess alcohol dependence in individuals in a Chinese community setting. A secondary aim was to examine the association between alcohol dependence and hypertension.

## Methods

### Study design and participant selection

This population-based, cross-sectional survey was conducted in Xuzhou, Jiangsu, China, from February 2013 to June 2013. The Probability proportional to size was used to select the sample from all 11 regions in Xuzhou city. First, each region selected five subdistricts/townships in urban/rural areas. Second, each subdistrict/township selected five communities/villages. Final, each household selected one person who had lived in their current residence for at least 5 years and was at least 18 years old with a Kish selection table. Participants who met one of the following criteria were excluded: (1) all patients with a previous diagnosis of hypertension were excluded because behavior control(such as eating less salt) might be a confounder; (2) members of the floating population or temporary residents; (3) individuals with a disability or other severe diseases (such as Kidney disease, Coronary heart disease and stroke etc). Participants were interviewed face-to-face by trained investigators on the day of their regular medical appointments. A total of 39,454 people were selected and 36,157 participated in the survey. The overall response rate was 91.6%.

Each participant was measured three times blood pressure in a sitting position after 5 min of rest by trained and certified observers according to the recommended procedure by the American Heart Association [16]. The participant need to rest 2 min after each measurement, and then do a next measurement. In addition, participants were asked to avert drinking, smoking, using coffee or tea, and physical Training for at least 30 min before their blood pressure measurement. The average of the three readings on consequent 3 days would be used for analysis. One of three cuff sizes (regular adult, large or thigh) was chosen with a standardized mercury sphygmomanometer according to the circumference of the participant’s arm [16]. Hypertension was defined as either an average systolic blood pressure ≥ 140 mmHg or an average diastolic blood pressure ≥ 90 mmHg on at least three separate occasions in a seated position in the right arm [17]. A multistage stratified cluster sampling method based on location, economic characteristics and population distribution was used to obtain prevalence estimates with appropriate precision and adequate representation. Finally, a sample of 36,157 residents of 22 districts in five counties of Xuzhou was interviewed.

Age, sex, body mass index (BMI), marital status, average monthly income, level of education, cigarette smoking, physical activity and family history of hypertension were recorded using a standard self-designed questionnaire. Body height, weight and blood pressure were measured using standard equipment and methods, and BMI was defined as underweight (< 18.5 kg/m^2^), normal weight (18.5 kg/m^2^ to 23.9 kg/m^2^), overweight (24.0 kg/m^2^ to 27.9 kg/m^2^) and obese (≥28.0 kg/m^2^) [18]. Marital status was categorized as single, widowed, married and others (i.e., divorced or separated). Education was categorized as below primary school and primary school, middle school, college and above college level. Cigarette smoking was defined as having smoked at least 100 cigarettes over the lifetime and smoking cessation was defined as having stopped smoking for more than 6 months. Physical activity was defined as participating in moderate or vigorous activity for no less than 30 min per day at least 3 days a week and was measured using the validated Chinese short version of the International Physical Activity Questionnaire [19]. Alcohol drinking was defined as the consumption of at least 30 g alcohol per week for 1 year or more. The Michigan Alcoholism Screening Test (MAST) [20] was used to estimate the level of alcohol dependence. The MAST consists of 25 questions with “yes/no” responses and detects alcohol problems over the last 12 months. Possible item scores are 0, 1, 2 or 5 points. The total MAST score varies between 0 and 53 points [20]. Higher scores indicate greater alcohol dependence. A score of 0 indicates no alcohol dependence, 1–4 indicates low alcohol dependence, 5–6 scores indicates light alcohol dependence, 7–25 indicates mild alcohol dependence, 26–39 indicates moderate alcohol dependence and 40–53 indicates severe alcohol dependence. Participants with a MAST score of at least 5 were considered to be alcohol dependent [20]. The sensitivity and specificity of this cutoff point are, respectively, 0.92 and0.83 [21].

All the investigators and staff members were carefully trained using standardized protocols for the questionnaires, blood pressure measurements and anthropometric measurements.

### Statistical analyses

All statistical analyses were conducted using SPSS 18.0 (IBM Corporation, Armonk, NY, USA). Data were entered twice independently using EpiData 3.1 and checked for errors and inconsistencies to enhance accuracy. Enumeration data were compared using chi-square and quantitative data were compared using t-tests. Spearman’s rank correlation was used to estimate the relationship between alcohol dependence and newly detected hypertension. Logistic regression analysis was performed to measure the probability that alcohol dependence predicts hypertension with adjusted risk factors including age, sex, BMI, marital status, average monthly income, level of education, cigarette smoking and physical activity. All confidence intervals (CIs), estimations and tests used a two-sided error rate of 5%. Continuous variables were described using mean ± standard error or median and quartiles.

## Results

There were 39,454 participants and 3297 individuals declined to take part. The mean age of participants was 45.5 ± 16.6 years, and 48.40% were male. The mean age of non-respondents was 45.1 ± 16.8 years, and 49.1% were male. 6178 participants (18.8%) who had the history of hypertension were excluded. There were no significant differences between participants and non-respondents (*P* > 0.05). The characteristics of the participants are shown in Table 1.

**Table 1:** Baseline characteristics and risk factors for hypertension in participants by level of alcohol dependence

Variables	Number	Non-drinkers (%)	Levels of alcohol dependence (%)				χ^2^	*P* value
			Non-dependence	Low	Light	Medium^a^		
Gender								
Male	17,501	12,903(73.73)	744(4.25)	1244(7.11)	1969(11.25)	641(3.66)	4412.82	< 0.01
Female	18,656	18,255(97.85)	77(0.41)	111(0.59)	175(0.94)	38(0.20)		
Age								
18~	4619	4262(92.27)	72(1.56)	97(2.10)	150(3.25)	38(0.82)	315.72	< 0.01
25~	6485	5630(86.82)	158(2.44)	238(3.67)	373(5.75)	86(1.33)		
35~	7579	6393(84.35)	204(2.96)	346(4.57)	455(6.00)	181(2.39)		
45~	6373	5324(83.54)	162(2.54)	267(4.19)	449(7.05)	171(2.68)		
55~	6154	5189(84.32)	112(1.82)	284(4.61)	430(6.99)	139(2.26)		
65~	3202	2789(87.10)	79(2.47)	87(2.72)	198(6.18)	49(1.53)		
75~	1745	1571(90.03)	34(1.95)	36(2.06)	89(5.10)	15(0.86)		
Area								
Urban	9390	8070(85.94)	285(3.04)	405(4.31)	502(5.35)	128(1.36)	68.74	< 0.01
Country	26,767	23,088(86.26)	536(2.00)	950(3.55)	1642(6.13)	551(2.06)		
Marital status								
Single	4076	3772(92.54)	58(1.42)	79(1.94)	126(3.09)	41(1.01)	265.46	< 0.01
Widowed	1933	1768(91.46)	22(1.14)	41(2.12)	75(3.88)	27(1.40)		
Married	30,026	25,505(84.84)	741(2.47)	1231(4.10)	1940(6.46)	609(2.03)		
Others	107	98(91.59)	0	4(3.74)	3(2.80)	2(1.87)		
Level of education								
Primary school or below	12,666	11,295(89.18)	182(1.44)	355(2.80)	644(5.08)	190(1.50)	187.89	< 0.01
Middle school	13,516	11,356(84.02)	349(2.58)	597(4.42)	905(6.70)	309(2.29)		
High school	6134	5200(84.77)	178(2.90)	252(4.11)	389(6.34)	115(1.87)		
College or above	3832	3298(86.06)	112(2.92)	151(3.94)	206(5.38)	65(1.70)		
Physical activity								
Lack	2884	2486(86.20)	63(2.18)	107(3.71)	156(5.41)	72(2.50)	30.63	< 0.01
Low	13,912	11,900(85.54)	349(2.51)	550(3.95)	797(5.73)	316(2.27)		
Medium	7444	6377(85.67)	196(2.63)	286(3.84)	449(6.03)	136(1.83)		
Heavy	522	423(81.03)	10(1.92)	24(4.60)	54(10.34)	11(2.11)		
Average monthly income								
< 350	2087	1838(88.07)	33(1.58)	68(3.26)	117(5.61)	31(1.49)	71.7	< 0.01
350~	8070	7042(87.26)	133(1.65)	261(3.23)	489(6.06)	145(1.80)		
700~	6338	5506(86.87)	123(1.94)	228(3.60)	358(5.65)	123(1.94)		
1000~	14,477	12,307(85.01)	391(2.70)	618(4.27)	885(6.11)	276(1.91)		
2000~	3493	3028(86.69)	94(2.69)	124(3.55)	182(5.21)	65(1.86)		
3000~	1657	1408(84.97)	45(2.72)	56(3.38)	109(6.58)	39(2.35)		
Smoking								
Often	4949	2397(48.43)	352(7.11)	663(13.40)	1119(22.61)	418(8.45)	9030.09	< 0.01
On occasion	1863	1130(60.65)	121(6.49)	197(10.57)	325(17.44)	90(4.83)		
Quit	262	137(52.29)	23(8.78)	27(10.31)	63(24.05)	12(4.58)		
No smoking	29,076	27,488(94.54)	324(1.11)	468(1.61)	637(2.19)	159(0.55)		
BMI^b^								
Underweight	1060	983(92.74)	9(0.85)	19(1.79)	36(3.40)	13(1.23)	244.94	< 0.01
Normal	20,316	17,913(88.17)	415(2.04)	662(3.26)	1009(4.97)	317(1.56)		
Overweight	11,806	9792(82.94)	316(2.68)	532(4.51)	893(7.56)	273(2.31)		
Obese	2800	2318(82.79)	77(2.75)	137(4.89)	194(6.93))	74(2.64)		

Caution: ^a^Twenty-five participants were considered to have heavy alcohol dependence, but were classified as having medium alcohol dependence owing to the relatively small sample size. ^b^*BMI* body mass index

A total of 4999 participants were classified as alcohol drinking. The rate of alcohol drinking was 13.83% (4999/36157). The rate of alcohol drinking of men was higher than that of women(χ^2^ = 4677.74, *P* < 0.01). A total of 4178 participants (11.56%) met the criteria of alcohol dependence. There were significant differences in alcohol dependence for sex, age, areas, marital status, average monthly income, education level, physical activity level and body type (all *P* < 0.01) (Table 1). The prevalence of newly detected hypertension in non-drinking, drinking without alcohol dependence, the low alcohol dependent group, the light alcohol dependent group and the moderate alcohol dependent group was 9.08% (2829/31158), 8.65% (71/821), 12.25% (166/1355), 12.22% (262/2144) and 13.84% (94/679), respectively. The results from the chi-square analysis for linear trends indicated that participants with greater alcohol dependence had a significantly increased risk of hypertension (χ^2^ = 48.66, *P* < 0.01). Spearman’s rank correlation analysis also showed that alcohol dependence was positively correlated with systolic (*r* = 0.071) and diastolic (*r* = 0.077) blood pressure (all *P* < 0.01). Individuals who consumed alcohol had significantly increased risk of hypertension compared with non-drinking (odds ratio [OR]: 1.21, 95% CI: 1.09 − 1.37, *P* < 0.01), after adjusting for age, sex, BMI, marital status, average monthly income, level of education, family history of hypertension, cigarette smoking, physical activity and area.

Of the 36,157 participants, 3422 had hypertension and the prevalence was 9.46%. Participants’ systolic blood pressure and diastolic blood pressure were 120.3 ± 11.6 mmHg and 77.0 ± 7.7 mmHg, respectively. The Table 2 show the blood pressure in alcohol dependence subgroups. Twenty-five participants were considered to be heavily dependent on alcohol and were classed as moderately alcohol dependent because of the relatively small sample size. Significant associations were found between different levels of alcohol dependence and systolic or diastolic blood pressure (all *P* < 0.01).

**Table 2:** The blood pressure in subgroup alcohol dependence

group	systolic blood pressure(mm Hg)	diastolic blood pressure(mm Hg)
non-drinker group	120.0 ± 11.6	76.8 ± 7.7
alcohol non-dependent	121.4 ± 10.3	78.0 ± 6.8
low alcohol dependence group	122.1 ± 11.4	78.2 ± 7.5
light alcohol dependence group	122.1 ± 11.6	78.4 ± 7.9
medium alcohol dependence group	123.1 ± 11.7	79.5 ± 7.7
total	120.3 ± 11.6	77.0 ± 7.7

Multiple logistic regression models indicated that alcohol dependence was an independent risk factor for hypertension after adjusting for confounders including age, sex, BMI, marital status, average monthly income, level of education, family history of hypertension, cigarette smoking, physical activity and area; the total OR for alcohol dependence was 1.37, 95% CI: 1.19–1.71. For low alcohol dependence, OR: 1.44, 95% CI: 1.14–1.81, *P* < 0.01; for light alcohol dependence, OR: 1.35, 95% CI: 1.11–1.64, *P* < 0.01; for medium alcohol dependence, OR: 1.83, 95% CI: 1.40–2.41, *P* < 0.01. These results are shown in Table 3.

**Table 3:** Logistic regression analysis of the association between alcohol dependence and hypertension

Variables	*β*	*SE*	*Wald*	*P* value	*OR*	95% *CI*
Model 1						
Nonalcoholic			51.94	< 0.01	1	
Independence	−0.05	0.13	0.18	>0.05	0.95	0.71~ 1.21
Low dependence	0.34	0.09	15.48	< 0.01	1.40	1.12~ 1.65
Light dependence	0.33	0.07	23.30	< 0.01	1.39	1.16~ 1.60
Medium dependence	0.48	0.11	17.76	< 0.01	1.61	1.13~ 2.01
Model 2						
Nonalcoholic			29.78	< 0.01	1	
Independence	−0.03	0.16	0.04	>0.05	0.97	0.70~ 1.34
Low dependence	0.37	0.12	9.63	< 0.01	1.44	1.14~ 1.81
Light dependence	0.30	0.10	9.18	< 0.01	1.35	1.11~ 1.64
Medium dependence	0.61	0.14	18.97	< 0.01	1.83	1.40~ 2.41

Caution: Model 1, univariate logistic regression; model 2, multiple logistic regression after adjusting for confounding factors including age, sex, BMI, marital status, average monthly income, level of education, cigarette smoking, physical activity, and area

Abbreviations: *SE* standard error, *OR* odds ratio, *CI* confidence interval

## Discussion

This study produced three main findings. First, alcohol dependence was associated with increased odds of having hypertension. Second, the association between alcohol dependence and hypertension increased with greater levels of alcohol dependence. Third, the alcohol drinking rate and the alcohol dependence rate were 13.83% and 11.56%, respectively.

As the results indicate, the alcohol drinking rate of participants living in Xuzhou was lower than the average drinking rate in China [22] (21.10%), Singapore [23] (14.3%) and six European countries [3] (64.4%). However, the alcohol dependence rate was higher than the dependence rate of residents living in rural areas of Guangxi, China [24] (5.81%) and some European countries [3] (5%). This discrepancy may be because of differences in study design, sampling size, region and the clinical features of the study population, but could also be a result of ethnic group differences, as many studies have showed that ethnic groups differ in prevalence of alcohol consumption and alcohol dependence [25].

Numerous epidemiologic studies have been conducted to clarify the association between alcohol consumption and hypertension. However, the resultant findings are not consistent. Some studies have shown that alcohol consumption increases the risk of hypertension. For example, Li et al. reported that non-drinking Chinese adults had a lower prevalence of hypertension than current drinking [26]. Wang and colleagues found that only heavy drinking was associated with hypertension [27]. Other researchers have found a linear relationship between alcohol and blood pressure only for men [28, 29]. Zatu et al. found that systolic blood pressure and diastolic blood pressure were positively correlated with self-reported alcoholic intake (*r* = 0.513, *P* = 0.023; *r* = 0.571, *P* = 0.005, respectively) [30], which is consistent with our findings.Other studies have not identified an association between alcohol use and hypertension. For instance, Kavishe et al. [31] demonstrated that alcohol consumption was not associated with hypertension prevalence. Shanthirani et al. also failed to find any relationship between alcohol use and hypertension [32]. In addition, some studies have shown a protective effect of alcohol on hypertension [33, 34]. For example, Chataut and colleagues reported that drinking show a decreased risk of hypertension [33]. However, the protective effect of alcohol on hypertension was only found for light and/or moderate alcohol users [15]. Moreover, this relationship only occurs for light-to-moderate alcohol consumption in women by other research demonstrated [29, 34].

A meta-analyses of 16 prospective studies with 227,656 subjects (33,904 men and 193,752 women) demonstrated that alcohol use is consistently associated with increased hypertension risk. This meta-analysis showed that, compared with non-drinking, heavy alcohol consumption in men is associated with increased risk of hypertension, and low and moderate alcohol consumption is associated with a trend toward increased risk of hypertension. Among women, the meta-analysis indicated protective effects at < 10 g/d (RR: 0.87; 95% CI: 0.82–0.92; *P* < 0.001) and a trend toward decreased risk of hypertension with alcohol consumption 11 to 20 g/d (RR: 0.9; 95% CI: 0.87–1.04; *P* = 0.17), whereas a significantly increased risk of hypertension was associated with heavy alcohol consumption of 21 to 30 g/d (RR: 1.16; 95% CI: 0.91–1.46; *P* = 0.23) and 31 to 40 g/d (RR: 1.19; 95% CI: 1.07–1.32; *P* = 0.002) [14]. These findings are supported by our results, which demonstrated that alcohol dependence was significantly associated with increased risk of developing hypertension. The contrasting findings for the association between alcohol consumption and hypertension might be the use of different tools for the measurement of alcohol dependence as well as different methods for BP measurement.

The multiple logistic regression models showed that alcohol dependence was an independent risk factor for hypertension even after adjusting for confounders, and the odds ratios gradually increased with greater alcohol dependence. However, the biological mechanism underlying the development of hypertension in association with alcohol use remains unclear. Several hypotheses have been suggested to explain this process: (1) alcohol influences the function of the vascular endothelium by activating the renin–angiotensin–aldosterone system and inhibiting vasodilation [35]; (2) the inhibition of catalytic activities in 11β-hydroxysteroid dehydrogenase type caused by alcohol results in an increased level of plasma cortisol and a decreased level of aldosterone, which promote the development of hypertension [36, 37]; (3) alcohol drinking may lead to insulin resistance [38]; (4) ethanol consumption increase in sympathetic nervous system activity, stimulation of the renin-angiotensin-aldosterone system, an increase of intracellular Ca2+ in vascular smooth muscle, increased oxidative stress and endothelial dysfunction [39]; (5) alcohol increases the concentration of Ca2^+^ and Na^+^ in vascular smooth muscle cells and causes vasoconstriction [40]; and (6) alcohol results in endothelial dysfunction and synthesis inhibition of nitric oxide (NO) [41, 42]. However, confirmatory evidence for these theories is lacking and further studies are required.

Although the confounding factor that behavior control might influence living habits was adjusted by excluding patients who had already been diagnosed before our study, some limitations should be noted. First, this was a cross-sectional study; therefore, we could establish only an association between variables, not the presence of causation. Further randomized controlled or cohort studies are required. Second, information about alcohol dependence was self-reported by participants, not objectively measured. Therefore, the possibility of recall bias cannot be completely rejected. Third, dietary habits were not considered in this study; drinking generally consume a salty and unbalanced diet [43], which may increase blood pressure.

## Conclusions

In summary, our study showed that the prevalence of alcohol dependence is a higher in the Chinese city of Xuzhou City, and alcohol dependence was associated with increased hypertension risk. Thus, one of the priorities should be given to strengthening health education and precautionary and family health measures for excessive drinking. Further well-designed studies are needed to strengthen our conclusions.

## Abbreviations

BMI: Body mass index
CI: Confidence interval
MAST: Michigan Alcoholism Screening Test
OR: Odds ratio
SE: Standard error
